# Clinical features and prognosis of pulmonary enteric adenocarcinoma: A retrospective study in China and the SEER database

**DOI:** 10.3389/fonc.2023.1099117

**Published:** 2023-03-27

**Authors:** Qike Wang, Lu Zhang, Huahua Li, Linlin Liu, Xu Sun, Huaimin Liu

**Affiliations:** Department of Integrated Chinese and Western Medicine, Affiliated Cancer Hospital of Zhengzhou University and Henan Cancer Hospital, Zhengzhou, China

**Keywords:** pulmonary enteric adenocarcinoma, treatment, prognosis, survival analysis, real word data

## Abstract

**Objective:**

Pulmonary enteric adenocarcinoma (PEAC) is a rare subtype of pulmonary adenocarcinoma that lacks effective treatment. The purpose of this research was to investigate the clinical characteristics, treatment, and prognosis of PEAC, as well as the impact of relevant factors on survival, thus providing a reference for the clinical management of patients with this disease.

**Methods:**

For this study, we gathered clinical data from 26 patients with PEAC in the Affiliated Cancer Hospital of Zhengzhou University from June 2014 to June 2021. We used SEER*Stat software V8.3.5 to download the PEAC patients from the Surveillance, Epidemiology, and End Results (SEER) database. In total, 20 patients were identified. Clinical data, including general information, imaging findings, and treatment protocols, were obtained, together with a follow-up of disease regression. The relevant clinical data were then analyzed.

**Results:**

It included 12 males and 14 females out of 26 patients from China, whose mean age was (62.73 ± 11.89) years; 20 were in the lower lung, 11 were stage I-II, and 15 were stage III-IV. Five had EGFR mutations, and four had KRAS mutations. In terms of treatment, patients with stage I-II were primarily treated by surgery, and patients with stage III-IV were treated mostly by chemotherapy. We extended the follow-up date to January 2022. On completion of the follow-up visit, 11 patients died, and the remaining 15 patients survived. The overall survival (OS) of 26 patients was 2.0-76.0 months, while the mean was 53.1 months, and the median OS (mOS) was 38.0 months (95% CI:1.727-74.273). In the case of progression-free survival (PFS) times, it was 2.0-76.0 months, with a mean PFS of 31.0 months and a median PFS (mPFS) of 8.0 months (95% CI:4.333-11.667). The PFS of the 15 patients in stage III-IV was 2.0-17 months, while the mean PFS was 6.5 months and the mPFS was 6.0 months (95% CI:4.512-7.488). Out of the 20 patients identified in the SEER database, the average age was 69.9 years, with 14 males and 6 females. Of these patients, 8 were diagnosed with stage I-II, while the remaining 11 were diagnosed with stage III-IV. 10 underwent surgery, 4 received radiation therapy, and 9 received chemotherapy. The mean OS of the 20 patients was 67.5 months, mOS was 28.0 months (95% CI: 9.664- 46.336). For patients diagnosed with stage III-IV, the mean OS was 14.8 months and mOS was 20 months (95% CI: 4.713-35.287).

**Conclusion:**

PEAC is rare, and the prognosis is determined mainly by the stage; patients who undergo surgery in stage I-II have a better prognosis.

## 1 Introduction

Pulmonary enteric adenocarcinoma (PEAC) is a rarely seen pathological subtype of lung adenocarcinoma. Tsao and Fraser defined PEAC creatively for the first time in 1991 (1). The disease has subsequently been reported and researched by scholars in clinical practice. In 2015, the World Health Organization classified pulmonary enteric adenocarcinoma as one of the types of lung adenocarcinoma. It is defined as a primary lung tumor that shares histological and immunohistochemical features with colorectal cancer. The key diagnostic point of PEAC is to exclude gastrointestinal metastases, which should first contain >50% of the features similar to the cellular structure of colon adenocarcinoma, that is, tall columnar cells, with eosinophilic cytoplasm, formed into irregularly shaped glands, part of which may comprise necrotic material. Secondly, for at least one of the immunohistochemical markers of intestinal differentiation (CDX2: caudal type homeobox 2, CK20: cytokeratin 20, or MUC2: mucin 2), it is positive. It often expresses CDX2 and cytokeratin 7 (CK7), in contrast to thyroid transcription factor-1 (TTF-1) and CK20, which are usually negative, but any combination is possible (2). The morbidity of PEAC is not high; hence, most of the studies on the disease have been case reports over the years, leaving its prognosis unclear. There are no specific treatment guidelines for PEAC, and the current treatment strategy is similar to that for non-small cell lung cancer (NSCLC). In the early stages, surgery is the mainstay of treatment, while chemotherapy and radiotherapy may be used in the later stage. However, targeted therapy and immunotherapy are less commonly discussed.

In this study, we summarized and analyzed the clinical features and prognostic factors of this disease in our study center and the SEER database, seeking to improve the understanding and treatment of PEAC.

## 2 Materials and methods

### 2.1 Study population

Twenty-six patients were recruited for the project between June 2014 and June 2021.

#### 2.1.1 Inclusion criteria

Patients with PEAC diagnosed by pathological examination.

#### 2.1.2 Exclusion criteria

(1) Patients with concurrent primary malignancies of other systems within five years; (2) Combination of refractory or other serious life-threatening diseases. The research was reviewed and approved by the Ethics Committee of the Cancer Hospital of Zhengzhou University. The written informed consent waived by the ethics committee. Ethical Review No. (2019083002).

This study also collected PEAC patients from the SEER database. The samples were selected by downloading SEER Research Plus Data, 12 Registries, Nov 2021 Sub (1992-2019) from the SEER database using SEER*Stat software V8.3.5. Inclusion criteria: (1) Pathological diagnosis of PEAC (International Classification of Diseases for Oncology ICD-O-8144); (2) Primary focus limited to lung {Site and Morphology. Site recode ICD-O-3/WHO 2008} =‘ Lung and Bronchus’ AND {Site and Morphology. ICD-O-3 Hist/Behav, malignant} =8144/3: Adenocarcinoma, intestinal type’. Twenty patients were included in the study.

### 2.2 Variable collections

Clinical information of the patient is recorded, including age, gender, tumor site, family history, personal history, clinical symptoms, imaging findings (including tracheoscopy, CT, MRI, PET/CT imaging, etc.), tumor stage, tumor markers, surgical situation, post-operative pathology results, recovery after surgery, treatment programs, as well as disease regression.

### 2.3 Follow-up

Follow-up was done by reviewing medical records and communicating *via* phone, with a deadline of January 2022 or patient death. Overall survival (OS) is considered to be the time interval from the diagnosis of PEAC to death or the end of follow-up, while progression-free survival (PFS) is considered to be the time to disease progression or the time to the end of follow-up.

### 2.4 Statistical methods

①The collected clinical data were summarized and statistically described. ②The statistical analysis was carried out using SPSS 26.0 software: the Kaplan-Meier method was selected to compute the median survival, and graph survival curves; the Log-rank test was applied to make comparisons of between-group differences in survival curves, and univariate analysis was performed. A multiple-factor analysis of survival using a Cox proportional risk regression model (Cox model) to compare prognostic influences. For our test, a P value < 0.05 was accepted as statistical significance.

## 3 Results

### 3.1 Clinical characteristics

**Table 1** summarizes the PEAC data for the 26 patients, including 46.2% males and 53.8% females. Their age range was 46 to 86 years, with a mean age of 62.73 years. The primary tumor was located on the left lung in 11 cases (42.3%), on the right lung in 14 cases (53.8%), in the lower lung in 20 cases (76.9%), as well as in the upper lung in three cases (11.5%). It was observed that a family history of cancer in 8 cases (30.8%), including three cases of direct relatives with a history of lung cancer. There were six cases with a smoking history (23.1%). The initial symptoms included cough, sputum, hemoptysis, shortness of breath or chest pain, etc. Moreover, 11 cases were in stage I-II (42.3%), 15 in stage III-IV (57.7%), while stage IV patients showed mainly lung, bone, liver, distant lymph nodes, and adrenal metastases. Besides, five cases (19.2%) had epidermal growth factor receptor (EGFR) mutation, and four (15.4%) had Kirsten rat sarcoma viral oncogene homolog (KRAS) mutation. In terms of tumor markers, the Carcinoembryonic antigen (CEA) positivity rate was 55.6% within the range of 0.48 - 370.3 ng/mL, the neuron-specific enolase (NSE) positivity rate was 33.3% in the range of 9.99 - 23.46 ng/mL, and the cytokeratin 19 fragment (CYFRA211) positivity rate was 72.2% wide (1.53 - 130 ng/mL). In terms of treatment, 12 cases received first-line surgery, 19 received chemotherapy, two received radiotherapy, and three received targeted therapy.

**Table 1:** Clinical characteristics of 26 patients from China.

Variables	N=26
Age (years)	62.73 ± 11.89
≤65	15(57.7)
>65	11(42.3)
Gender, n (%)	
Male	12(46.2)
Female	14(53.8)
Clinical symptoms, n (%)	
Coughing and/or coughing up sputum	6(23.1)
Coughing up blood	6(23.1)
Shortness of breath and/or wheezing	4(15.4)
Chest or back pain	4(15.4)
Fever	1(3.8)
No symptom	4(15.4)
Axillary mass	1(3.8)
Family history of cancer, n (%)	8(30.8)
Smoking history, n (%)	6(23.1)
History of alcohol intake, n (%)	4(15.4)
Primary site, n (%)	
Upper lobe	3(11.5)
Middle lobe	1(3.8)
Lower lobe	20(76.9)
Laterality, n (%)	
Left	11(42.3)
Right	14(53.8)
Double-sided	1(3.8)
Clinical stage, n (%)	
Stage I	5(19.2)
Stage II	6(23.1)
Stage III	3(11.5)
Stage IV	12(46.2)
Genic mutation, n (%)	
EGFR	5(19.2)
KRAS	4(15.4)
TP53	2(7.7)
ERBB2	3(11.5)
Peripheral Blood Biomarkers, n (%)	
CEA, (0-4.7ng/mL)	10/18(55.6)
NSE, (0-16.3ng/mL)	6/18(33.3)
CYFRA 21.1, (0-3.3ng/mL)	13/18(72.2)
CA 19.9, (0-27U/mL)	2/2(100)
First-line treatment	
Surgery, n (%)	12(46.2)
Radiation, n (%)	3(11.5)
Chemotherapy, n (%)	19(73.1)
Targeted therapy, n (%)	3(11.5)
Untreated	1(3.8)
Death, n (%)	11(42.3)

EGFR, epithelial growth factor receptor; KRAS, Kirsten rat sarcoma viral oncogene homolog; TP53, tumor protein p53; ERBB2, human epidermal growth factor receptor 2; CEA, carcinoembryonic antigen; NSE, neuron-specific enolase; CYFRA 21.1, cytokeratin 19 fragment.

### 3.2 Prognostic analysis

**Figure 1:**
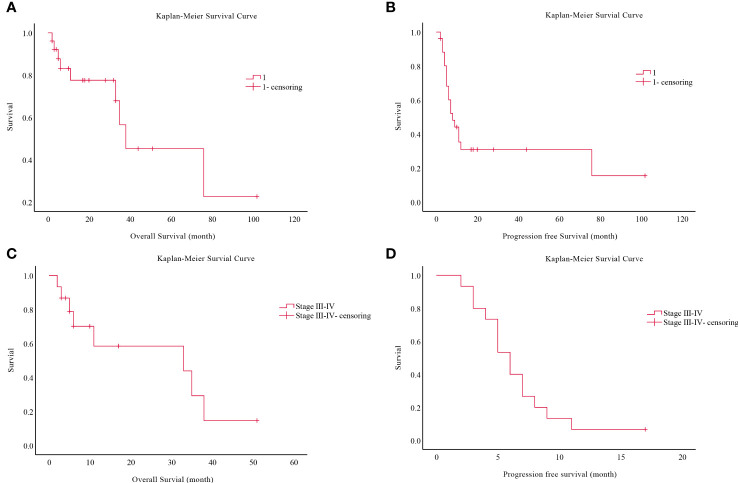
Survival analysis of 26 patients from China. **(A)** Overall survival analysis of 26 patients. **(B)** Progression free survival analysis of 26 patients. **(C)** Overall survival analysis of stage III-IV. **(D)** Progression free survival analysis of stage III-IV.

Eleven of the 26 patients had passed away when the study ended, nine of disease progression, one of pulmonary infection, and one of post-operative bronchial stump fistula **Figure 1**. Despite this, four cases were lost to follow-up with no OS obtained. The OS for 26 patients was 2.0-76.0 months, with a mean OS of 53.1 months and a median OS (mOS) of 38.0 months (95% confidence interval (CI):1.727-74.273). In addition, PFS was 2.0-76.0 months, the average PFS was 31.9 months, and the median PFS (mPFS) was eight months (95% CI:4.333-11.667). After this, we performed a survival analysis of 15 patients in stages III-IV. We found that OS was 2-38.0months, for a mean OS of 25 months and a mOS of 33 months (95%CI: 0.0-83.085). Meanwhile, PFS was 2-11.0 months, with a mean PFS of 6.5 months and a mPFS of 6 months (95%CI: 4.512-7.488).

### 3.3 Univariate and multivariate prognostic analyses

To outline the factors associated with predicting the impact of PFS, we used univariate and multivariate COX regression models (**Tables 2**, **3**). Univariate analysis revealed that two factors, tumor stage and whether the surgery had been operated on, were associated with PFS. Naturally, we added variables with P<0.2 to the multivariate analysis; nevertheless, the differences in all variables were not statistically significant.

**Table 2:** Univariate analysis of PFS.

Variables	n	mPFS	t/χ^2^	P	95%CI
Age (years)			1.255	0.263	
≤65	15	11			5.14-16.86
>65	11	7			3.98-10.02
Gender			2.581	0.108	
Male	12	7			3.88-10.11
Female	14	11			0.0-42.78
Family history of cancer			1.74	0.187	
Yes	8	11			0.0-73.83
No	18	1			5.0-8.98
Smoking history			0.321	0.571	
Yes	6	5			0.83-9.17
No	20	8			3.62-12.38
History of alcohol intake			1.197	0.274	
Yes	4	5			–
No	22	8			2.56-13.44
Primary site			0.938	0.333	
Lower lobe	20	6			1.62-10.38
Others	6	9			4.71-13.29
Laterality			0.29	0.865	
Left	11	8			0.45-15.55
Right	14	9			5.00-13.00
Double-sided	1	7			–
Clinical stage			12.603	0.000	
Stage I-II	11	76			0.00-169.26
Stage III-IV	15	6			4.51-7.48
Surgery			9.762	0.002	
Yes	12	76			0.00-170.03
No	14	6			4.19-7.82
Radiation			2.514	0.113	
Yes	3	5			–
No	23	11			6.65-15.35
Chemotherapy			0.114	0.735	
Yes	19	8			4.80-11.20
No	7	76			–

**Table 3:** Multivariate Cox regression models associated with PFS.

Variables	B	SE	Wald	HR	95%CI	P
Gender	-0.605	0.539	1.259	0.546	0.190-1.571	0.262
Family history of cancer	0.119	0.691	0.030	1.126	0.291-4.362	0.863
Clinical stage	1.030	1.321	0.609	2.802	0.211-37.292	0.435
Surgery	-1.382	1.125	1.510	0.251	0.028-2.275	0.219
Radiation	0.012	0.694	0.000	1.013	0.260-3.948	0.986

### 3.4 Treatment

**Figure 2:**
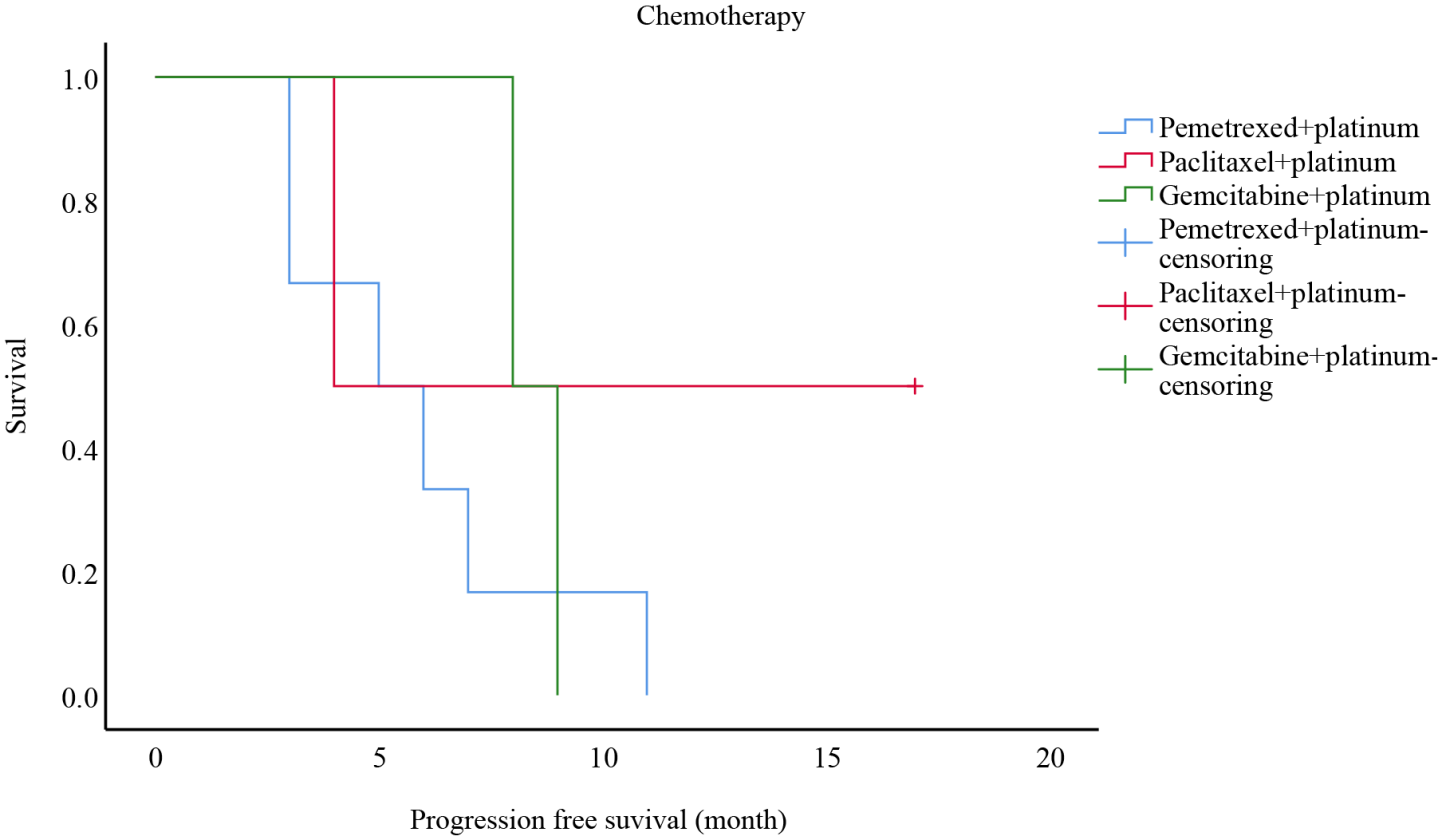
Progression free survival analysis of patients with different chemotherapy modalities in stages III-IV from China.

Ten of these stage III-IV cases received only chemotherapy as first-line treatment: six of them received pemetrexed + platinum-based regimens, two of them adopted paclitaxel-based + platinum-based protocols, and the remaining two were given gemcitabine + platinum-based chemotherapy. Survival analysis is shown in **Figure 2**. The mean PFS was 5.8 months with a mPFS of 5 months (95% CI: 1.399-8.601) for patients on the pemetrexed + platinum regimen, while the mean PFS was 10.5 months with a mPFS of 4 months (95%CI: -) among patients on the paclitaxel-like + platinum regimen, compared to 8.5 months with gemcitabine + platinum regimen with a mPFS of 8 months (95%CI: -), p=0.446. There was no statistically meaningful difference.

It was also discovered that two cases with EGFR exon 19 mutations in stage III-IV patients, one treated with pemetrexed + carboplatin + bevacizumab + gefitinib with a PFS of six months, the other with six cycles of paclitaxel + carboplatin followed by maintenance treatment with osimertinib, which did not progress by the end of follow-up with a PFS of 17 months.

### 3.5 Additionally, we analyzed 20 cases screened by the SEER database

**Figure 3:**
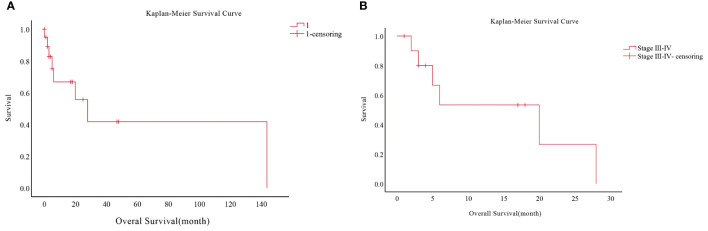
Survival analysis of 20 patients from the SEER database. **(A)** Overall survival analysis of 20 patients. **(B)** Overall survival analysis of stage III-IV patients.

Their age ranged between 39 and 86 years, giving a mean age of 69.9 years. A total of 14 of them were male, and six were female. The tumor was situated in the upper lung in nine cases, in the lower lung in eight cases, in the left lung in six, and in the right lung in thirteen. Regarding the tumor stage, eight were in stages I-II, and the remaining 11 were in III-IV. Concerning treatment, 10 underwent surgery, four radiotherapies, and nine chemotherapy (**Table 4**). We carried out a survival analysis of 20 patients, which suggested a mean OS of 67.5 months and a mOS of 28.0 months (95% CI: 9.664-46.336), yet the deletion rate was 60%, so the conclusions were for reference only. To understand the mortality of late-stage patients, we abstracted the OS of patients with stages III-IV and came up with a mean OS of 14.8 months and a mOS of 20 months (95% CI: 4.713-35.287) (**Figure 3**).

**Table 4:** Clinical characteristics of 20 patients from SEER database.

Variables	N=20
Age (years)	69.9 ± 10.26
≤65	5(25.0)
>65	15(75.0)
Gender, n (%)	
Male	14(70.0)
Female	6(30.0)
Race	
White	15(75.0)
Black	5(25.0)
Primary site, n (%)	
Upper lobe	9(45.0)
Middle lobe	2(10.0)
Lower lobe	8(40.0)
Unknown	1(5.0)
Laterality, n (%)	
Left	6(30.0)
Right	13(65.0)
Unknown	1(5.0)
Clinical stage, n (%)	
Stage I	6(30.0)
Stage II	2(10.0)
Stage III	4(20.0)
Stage IV	7(35.0)
Unknown	1(5.0)
Surgery, n (%)	10(50)
Radiation, n (%)	4(20.0)
Chemotherapy, n (%)	9 (45.0)

## 4 Discussion

In this study, the age range at diagnosis was 46-86 years, with a mean age of 62.73 years for the 26 patients with PEAC and a ratio of 6:7 for males to females. The age at diagnosis ranged from 39 to 86 years in the 20 patients with PEAC in the SEER database, with a mean age of 69.9 years. The ratio of males to females was 7:3. Raffaele Palmirotta et al. (3) identified 295 patients (116 males and 90 females) in articles published up to January 25, 2020. As a result of the analysis, the patients’ ages ranged from 25 to 81 years, with a mean of 63.24 years. It indicates that the average age of disease onset in patients is greater in the elderly, and the proportion of men and women with morbidity is unknown due to the small sample size. In the present investigation, the primary tumor occurrence ratio in the left lung to the right lung was 11:14, and that in the lower lung to the upper lung was 20:3. On the other hand, among the 20 patients in the SEER database, the ratio of the left lung to the right lung was 6:13, and that of the upper lung to the lower lung was 9:8. It was similar to the study by Haiyan Li et al. (4), who identified 103 patients with lesions mostly in the right lung tissue and a ratio of right lung lesions to left lung lesions of approximately 66:49. They found that lesions were mainly located in the upper lung, where the lesion sites were 59:40:5 in the upper lung: lower lung: middle lung. Our findings showed that the first symptoms of PEAC patients were cough, sputum, hemoptysis, chest tightness, shortness of breath, or chest pain. Moreover, six patients had a history of smoking. Remarkably, we found that eight of the 26 patients had a family history of cancer, with three having immediate family members who had lung cancer. The case report by Garajová et al. (5) recommended that their immediate family members were also diagnosed with PEAC, whereas the three immediate family members with lung cancer in this study were all deceased, so it is not known whether they were also PEAC or not. Thus, we included an immediate family member with a malignant neoplasm in the univariate analysis of the effect on PFS, although it was not statistically significant (p=0.187).

Five patients in this study had EGFR mutations, four of which were EGFR exon 19 mutations and one exon 21 mutation. KRAS mutation positivity was more frequent than tumor protein p53 (TP53) mutations and human epidermal growth factor receptor 2 (ERBB2/HER2). The most common gene mutations in the ten patients studied by Xie et al. (6) included TP53 (57%, 4/7) and KRAS (57%, 5/7) mutations. Tu, L. F. et al. (7) showed two patients with KRAS mutations, one patient with a KRAS missense mutation, and the other patient with a BRAC1 nonsense mutation and a KRAS missense mutation. It was also shown in a case report by Shimizu et al. (8) that PEAC carries rare BRAF G469V mutations. Wang et al. (9) discovered EGFR to be a critical driver mutation in PEAC, but its incidence was lower than that of classic lung adenocarcinoma, in contrast to ERBB2 and KRAS, which were more common in PEAC. Jurmeister et al. (10) observed TP53 mutations in 6 out of 7 samples and KRAS mutations in three cases. Jurmeister et al. (11) noted KRAS mutations in nine (60%) of 15 PEAC cases. Nottegar et al. (12) observed EML4-ALK rearrangements in 6/46 (13.0%) PEAC. 1/46 patients with PEAC had mutations in EGFR exon 19 (p.E746_S752) (2.2%), and 28 had the KRAS gene mutation at codon 12 (60.9%). There was no case showing BRAF mutation (0/46). According to Lin et al. (13), ALK/ROS1 point mutations were found in five cases (71.42%, 5/7) and MSH2/MSH6 point mutations in three cases (42.86%, 3/7). In contrast, all nine patients shown by Wang et al. (14) were EGFR and KRAS wild-type. Feng et al. (15) and Zhao et al. (16) found EGFR mutations in 13 (43.3%) of 30 patients, EGFR mutations in three (10.7%) of 28 patients, and KRAS mutations in ten cases 10/25 (40%), respectively. Nottegar et al. (17) assessed eight patients, 1/8 (12.5%) had both PIK3CA mutations and EML4-ALK translocations, while 4/8 (50%) had the KRAS gene mutation at codon 12. In contrast, NRAS, BRAF, and EGFR genes were all wildtypes. Accordingly, the most common mutations in PEAC are EGFR mutations and KRAS mutations, while TP53 mutations and ERBB2 amplifications, EML4-ALK rearrangements, and BRAF G469V mutations are less common.

To better identify the value of serum tumor markers in the diagnosis of PEAC, we examined the levels of tumor markers associated with lung cancer (CEA, NSE, and CYFRA 21.1) and the level of colorectal cancer-related tumor marker carbohydrate antigen (CA 19.9). Regrettably, CA 19.9 levels were detected at diagnosis in only two patients in this review, and one of them had serum CA 19.9 levels >1000 ng/ml. The positive rate of CEA and CYFRA 21.1 were more highly expressed than NSE expression in this report. Gu et al. (18) found that the positive rates of tumor markers CEA, CA19.9, and CA125 in PEAC were 71% (10/14), 50% (5/10), and 50% (5/10), respectively. Furthermore, Chen et al. (19) discovered that CEA and CA 19.9 were more abundant in primary cultured PEAC than CYFRA 21.1 and NSE. CA 19.9 was the richest expressed tumor marker, but NSE was barely expressed. CEA, CA 19.9, and CA125 were abnormally elevated in six PEAC cases shown by Tu et al. (7). CA 19.9 and CEA increased markedly over CA125. The highest values of CEA and CA 19.9 were 509 ng/mL and 1449.9 U/mL, separately. Both the NSE and CYFRA 21.1 were all normal. When diagnosing and monitoring lung cancer, physicians should look for CA 19.9 levels and lung cancer-related serum tumor markers. Eleven patients were in stages I-II and 15 patients were in stages III-IV at the time of diagnosis in this study. While 19 early-stage (stage I and II) patients and 9 stage III-IV patients were among the 28 patients with PEAC investigated by Zhao et al. (16). The most prevalent distant metastatic sites in patients with advanced stages were lung, bone, liver, distant lymph nodes, and adrenal metastases (6, 20), whereas skin and pancreatic metastases were rare (21, 22). Feng et al. (15) enrolled three patients (30%) in stages I and II and 27 (90%) in stages III-IV of the 30 patients included. Chen et al. (19) found 12 patients (67%) in early-stage (stage I-II) and 6 (90%) in stages III-IV of the 18 patients with PEAC. In the various small sample studies, the staging percentages at diagnosis were not found to have a regular pattern. Patients in stages I-II received mainly surgical treatment, while the main treatment in stages III-IV was chemotherapy, and radiotherapy and targeted therapy accounted for a small proportion. By the end of follow-up, 9 patients had died due to disease progression. It was found that 26 patients had an OS of 2.0-76.0 months, giving an average OS of 53.1 months as well as a mOS of 38.0 months (95% CI:1.727-74.273). Chen et al. (19) showed a median survival of 31 months (4-96 months) in 18 patients. The mOS of the 11 patients enrolled by Lin et al. (13) was nine months. Xie et al. (6) suggested that the median disease-free survival (DFS) of patients was 20.5 months (interquartile range, 16-28.3). Previous research has shown that the prognosis of patients with PEAC is directly associated with their clinical stage (23), with survival times ranging from 0 to 9 months for stage III or IV patients. In our research, the OS for stage III-IV patients was 2-38.0 months, the mean OS was 25 months, and the mOS was 33 months (95% CI: 0.0-83.085). 26 patients had a PFS of 2.0-76.0 months, with a mean PFS of 31.9 months as well as a mPFS of 8 months (95% CI: 4.333-11.667). A further analysis of stage III-IV patients then revealed that the PFS was 2-11.0 months, the mPFS was 6 months (95% CI: 4.512-7.488) and the mean PFS was 6 months. Furthermore, we analyzed 11 stages III-IV patients out of 20 cases from the SEER database, yielding a mean OS of 14.8 months and a mOS for 20 months (CI: 4.713-35.287). Lastly, the data from our study center were analyzed. We performed a univariate analysis of the factors of age, gender, family history of tumor, history of smoking, history of alcohol consumption, site, stage, and treatment, and concluded that tumor stage and whether surgery was associated with prognosis. No statistically significant differences were found in the multifactorial analysis, probably related to the small sample size of this study.

When it comes to systemic therapy, Teranishi et al. (24) reported a 68-year-old male with a stage IV B diagnosis, positive KRAS G12D mutation, and a tumor percentage score (TPS) of <1% for programmed cell death ligand 1 (PD-L1). Palliative radiotherapy and pembrolizumab + pemetrexed + carboplatin chemotherapy were administered, and the outcome was evaluated as partial response (PR). In comparison, Hu et al. (25) reviewed a 6 years old man with KRAS mutation in stage IV, who progressed rapidly after one cycle of paclitaxel + carboplatin + sintilimab. Tu et al. (7) reported that the four recipients received surgical treatment, curative knife treatment, and/or chemotherapy. The main chemotherapy regimens were pemetrexed + platinum (cisplatin or carboplatin) and paclitaxel + cisplatin, and the disease was controlled in all cases (efficacy evaluated as PR/stable disease (SD)). Patel et al. (20) covered a 60-year-old male patient treated with docetaxel + cisplatin after surgery. Six months later, the disease relapsed, then he received nivolumab, which remained effective for more than 14 months. As for targeted therapies and immunotherapy, scholars have described a case of a patient given icotinib (a first-generation EGFR TKI) for over 1.5 months and then treated with volutumab (an immunotherapy drug) for more than 9.5 months (13). As the pathology of PEAC is characterized by intestinal differentiation, it is feasible to treat it with chemotherapy for colorectal cancer. Lin et al. (26) described a 53 years old female in stage IV who was initially treated with the XELOX (capecitabine plus oxaliplatin), followed by disease progression. The pulmonary achieved partial remission after four cycles of chemotherapy with the TP (paclitaxel plus cisplatin) regimen. However, the supraclavicular response to the drug was poor. After two cycles of the FOLFIRI (5-fluorouracil, leucovorin, and irinotecan) regimen, the disease progressed again, and the patient has finally treated with the DP (docetaxel plus cisplatin) regimen after palliative surgery. Garajova et al. (5) presented a case of a 68 male patient who underwent surgery and was later found to have bone metastases, so he was treated with XELOX and bisphosphonates, the disease progressed after two cycles, and four months later, the patient had a recurrence of multi-site osseous metastases and received four cycles of carboplatin + pemetrexed, none of which prevented the progression of the tumor. Succeeding chemotherapy with doxorubicin stabilized the progression after two cycles. Likewise, the patient’s sister underwent a lobectomy. She was found to have stage IB PEAC, which progressed with pulmonary and adrenal metastases over 12 months. However, after receiving 6 cycles of carboplatin + pemetrexed and then pemetrexed alone, the disease stabilized. Qureshi et al. (27) reported a 61years old female with stable disease after four cycles of treatment with pemetrexed + carboplatin. According to Chen et al. (19), PEAC had a higher rate of TMB and MMR mutations than pulmonary adenocarcinoma (PAC). Manglaviti et al. (28) assessed PEAC data in ten cases with immune checkpoint inhibitors, yielding mPFS was 1.5 months (95% CI 0.2-2.8) and mOS was 17.3 months (95% CI 0.2-12.6). PR was 1 (10%) case, SD was 1 (10%) case, and PD was 8 (80%) cases. PEAC appears to have a poor response to immunotherapy, according to the research results. This suggests that PEAC is effectively treated with the pemetrexed/paclitaxel-based + platinum regimen, while it responds poorly with the XELOX/FOLFIRI regimen. In our center’s study, ten patients with stage III-IV I had chemotherapy alone as their first-line treatment: six of platinum-based chemotherapy, and two had gemcitabine + platinum-based chemotherapy. Mean PFS was 5.8 months for pemetrexed + platinum, mPFS was five months (CI: 1.399-8.601), mean PFS was 10.5 months for paclitaxel + platinum, mPFS was four months (CI: -), and mean PFS was 8.5 months for gemcitabine + platinum, mPFS was eight months (CI. -), P=0.446. There were two phase III-IV patients with EGFR exon 19 mutations, one of whom was given treatment with pemetrexed + carboplatin + bevacizumab + gefitinib with a PFS of six months and one treated with paclitaxel + carboplatin after six cycles and maintenance treatment with osimertinib, which did not progress to the end of follow-up with a PFS of 17 months.

## 5 Limitations

Due to the small sample size, the difference in prevalence between men and women is not known. A proportion of patients had a family history of malignancy, particularly lung cancer, and the relationship between family history and incidence was uncertain because of the limitations of the sample size. This was a retrospective study and only 2 patients had examined CA 19.9 levels at the time of diagnosis, which prevented further assessment of the relationship between CA 19.9 and prognosis.

## 6 Conclusion

In conclusion, the onset of PEAC is most often seen in the elderly. Patients tend to seek treatment with chest complaints as the first symptom. The common genetic mutations are EGFR and KRAS mutations. CEA, CA 19.9, and CYFRA 21.1 levels must be monitored during diagnosis and follow-up. Surgery is often the mainstay of early treatment, while doublet chemotherapy with platinum-based is used in the late stages. Patients who can undergo surgery in the early stages have a better survival than those who cannot undergo surgery because of advanced cancer.

## Data availability statement

The original contributions presented in the study are included in the article/**Supplementary Material**. Further inquiries can be directed to the corresponding authors.

## Ethics statement

The studies involving human participants were reviewed and approved by Ethics Committee of the Cancer Hospital of Zhengzhou University. Written informed consent for participation was not required for this study in accordance with the national legislation and the institutional requirements.

## Author contributions

QW and XS contributed to conception and design of the study. QW and LZ contributed to the acquisition of the data. QW, LZ, HLi and LL contributed to the analysis and interpretation of data. QW wrote first draft of the manuscript. XS and HLiu contributed to the revision of the manuscript. All the authors made substantial contributions to this work and approved it for publication.
